# Genome and Comparative Transcriptome Analysis of Growth and Developmental Changes in the Pileus of the *Cyclocybe chaxingu*

**DOI:** 10.3390/jof12010063

**Published:** 2026-01-13

**Authors:** Liyuan Luo, Shiqi Wan, Yuling Zhou, Chezhao Wang, Chunyan Yang, Wenqi Huang, Ling Chen, Zhiting Yu, Sihan Li, Xiaolong Chai, Xinrui Liu

**Affiliations:** 1 Mycological Research Center, Fujian Agriculture and Forestry University, Fuzhou 350002, China; 2 College of Life Sciences, Fujian Agriculture and Forestry University, Fuzhou 350002, China

**Keywords:** *Cyclocybe chaxingu*, pileus expansion, pileus color, transcriptome, genome

## Abstract

*Cyclocybe chaxingu* is a well-known edible fungus in China, in which pileus size and color are key traits determining its commercial value. However, the molecular genetic mechanisms underlying the morphological development of its pileus remains limited at present. To address this, our study first completed the high-quality genome assembly of the monokaryotic strain Ag.c0002-1 of albino *C. chaxingu*, anchoring it to 13 chromosomes via Hi-C technology. The final genome size was 51.7 Mb with a GC content of 51.06%, and 11,332 protein-coding genes were annotated. Phenotypic observations and comparative transcriptome analyses were then conducted on the pilei of the brown cultivar Ag.c0067 and the white cultivar Ag.c0002 at the primordium, elongation, and mature stages. Phenotypic analysis revealed continuous pileus expansion accompanied by progressive color lightening in both cultivars during development. Comparative transcriptomic analyses revealed significant differences in gene expression patterns between the two cultivars across developmental stages. KEGG enrichment analysis indicated that pileus expansion is closely associated with pathways related to DNA replication, cell cycle of yeast, carbon metabolism, and carbohydrate digestion and absorption. Among these, differentially expressed genes involved in cell division tended to be downregulated, whereas genes associated with energy metabolism and substance transport were upregulated, providing the necessary energy and material support for pileus growth. Changes in pileus pigmentation were primarily associated with tyrosine metabolism, betalain biosynthesis, tryptophan metabolism, and melanogenesis pathways. Notably, the downregulation of tyrosinase genes and the upregulation of glutathione S-transferase genes during development may represent major molecular mechanisms underlying pileus color lightening. Overall, this study provides important insights into the molecular mechanisms regulating pileus development and pigmentation in *C. chaxingu*, while also offering valuable theoretical support for genetic analysis of basidiomycete morphogenesis and the molecular breeding of edible mushrooms.

## 1. Introduction

*Cyclocybe chaxingu* (synonym *Agrocybe cylindracea*) belongs to the phylum Basidiomycota, class Agaricomycetes, order Agaricales, family Strophariaceae, and genus *Cyclocybe*. The pileus is hemispherical, fleshy, smooth or wrinkled, ranging in color from white to brown, with the flesh typically white [1,2]. *C. chaxingu* is a common edible and medicinal fungus. It has attracted considerable attention due to its rich nutritional content, desirable organoleptic properties, and bioactive metabolites, which have been shown to exert effects on tumors and chronic diseases [3]. Research indicates that *C. chaxingu* is rich in protein and insoluble dietary fiber, while containing lower levels of fat and soluble dietary fiber. Furthermore, it contains 17 amino acids, with glutamic acid being the most abundant [4]. In terms of medicinal properties, *C. chaxingu* exhibits anti-aging, antioxidant, anti-cancer, and hypoglycemic effects [5,6,7,8,9]. In the commercial market, key marketable traits of *C. chaxingu* include fruiting body color, pileus size, and stipe length. Moreover, fruiting bodies at different developmental stages display distinct morphological features that affect both marketability and intrinsic quality. However, current research on the pileus expansion and color changes in *C. chaxingu* remains limited.

The genome constitutes the fundamental basis for decoding genetic information and plays a pivotal role in advancing research on species, trait inheritance mechanisms, functional gene identification, and molecular breeding [10]. Multiple genome assemblies of *C. chaxingu* (*Agrocybe cylindracea*/*Agrocybe aegerita*/*Agrocybe chaxingu*) have been reported, including strains AC9 (GenBank: MPNV00000000.1), MG21 (GenBank: QFEP00000000.1), AAE3 (GenBank: CACVBS000000000.1), and two sexually compatible mononucleate strains derived from AS-5 (BioProject accession number PRJNA1142205). Their genome sizes are 56.5 Mb, 56.3 Mb, 44.8 Mb, and 50.6 Mb and 51.7 Mb, respectively. Among these, the genomes of strains AC9, MG21, and AAE3 have not yet been assembled to telomere-to-telomere (T2T) resolution. In contrast, the genomes of the two mononucleate strains of AS-5 were assembled at the chromosome-level, each comprising 13 complete chromosomes [11,12,13,14]. The successful assembly of these genomes has established a solid foundation for in-depth investigations into the genetic mechanisms underlying fruiting body traits in *C. chaxingu*. However, published genome sizes of different strains exhibit considerable variation among strains with distinct phenotypic characteristics. Therefore, additional genomic data representing broader genetic diversity are still required to provide a more comprehensive reference for multi-trait analyses.

The pileus is not only the site for life reproduction, but also a structure whose size is a very important commercial trait. This size undergoes developmental changes regulated by complex mechanisms. Previous studies have revealed the key roles of various environmental factors and genes in this process. Studies on *Flammulina filiformis* have revealed that high CO_2_ concentrations affect the ubiquitin–proteasome system and cell cycle pathways, thereby inhibiting pileus expansion. Likewise, studies on *Coprinopsis cinerea* have shown that the transcription factor encoded by the *exp1* gene plays a crucial role in pileus expansion and autolysis, whereas the *dst1* gene promotes normal pileus differentiation and expansion by mediating light signaling and prevents pileus chlorosis [15,16,17].

Furthermore, the depth of the pileus color as another important commercial trait, is not only an intuitive phenotypic characteristic but also a crucial genetic marker that enables researchers to gain deeper insights into the fundamental mechanisms of developmental biology, genetics, and evolutionary processes [18]. In the field of edible and medicinal fungi, studies on the genetic mechanisms underlying pileus color, a representative chromatic trait, have achieved several significant advances. For instance, studies indicate that frameshift mutations in the phenylalanine deaminase 1 (*Fvpal1*) gene affect the color of *F. filiformis* fruiting bodies. These mutations cause the deletion of amino acids at positions 112 and 113, impairing the activity of *Fvpal1* and disrupting melanin synthesis, resulting in a white pileus phenotype in the mutant strain [19]. In *Auricularia cornea*, genetic maps were constructed to locate three pigment synthesis-related coding genes. The researchers found that fruiting body color is controlled by two pairs of alleles: when both pairs are dominant, the fruiting body exhibits a purple phenotype; when both pairs are recessive or one pair is recessive, the fruiting body exhibits a white phenotype [20,21]. Studies on the genetics of pileus color in *Hypsizygus marmoreus* revealed that the cytochrome P450 gene is a key regulator of color inheritance in this species [22]. Additionally, related research has been conducted on edible fungi such as *Pleurotus cornucopiae*, *Pleurotus salmoneostramineus*, and *Agaricus bisporus* [23,24,25].

The diversity of pileus traits has the potential to expand the varietal spectrum of *C. chaxingu*. At present, cultivated fruiting bodies of *C. chaxingu* in China predominantly exhibit two color types: brown and white. Among brown varieties, fruiting bodies with unopened pilei and darker coloration are generally favored for mushroom products, whereas in white varieties, fruiting bodies with a uniform and pure white appearance are preferred. After primordium formation, the fruiting bodies enter a rapid growth and development phase, characterized by continuous elongation of the stipe and expansion of the pileus, accompanied by distinct color changes—particularly in the central region of the pileus. Based on the chromosome-level assembly of the Ag.c0002-1 genome obtained using Hi-C and ONT sequencing data, this study utilized fruiting bodies from the white strain Ag.c0002 and the brown strain Ag.c0067 to observe pileus expansion and color variation during their developmental process. Furthermore, transcriptomic analyses were conducted to explore gene expression patterns at each developmental stage, aiming to identify candidate genes and metabolic pathways associated with pileus expansion and pigmentation. These findings provide a theoretical foundation for elucidating the genetic and molecular regulatory mechanisms underlying the phenotypic traits of *C. chaxingu*.

## 2. Materials and Methods

### 2.1. Strain Source

The strains used in this study included the brown heterokaryotic strain Ag.c0067, the white heterokaryotic strain Ag.c0002, and the white monokaryotic strain Ag.c0002-1 of *C. chaxingu*. The white monokaryotic strain Ag.c0002-1 was obtained from Ag.c0002 through protoplast monokaryotization. The protoplast mononuclearization procedure was performed as follows. Fresh mycelial tissue was collected from 100 mL of potato dextrose broth (PDB), washed twice with distilled water, and subsequently washed twice with isotonic buffer (0.7 M KCl, 10 mM CaCl_2_·2H_2_O). A total of 10–20 mL of 2% lywallzyme solution was then added, and the mixture was gently agitated at 30 °C and 60 rpm for 3 h. The released protoplasts were washed with isotonic buffer, filtered, and centrifuged at 2000× *g* for 2 min at 25 °C, after which the supernatant was discarded. The pellet was resuspended in Spheroplast Transformation Cocktail (20% sucrose, 50 mM CaCl_2_, 50 mM Tris-HCl) and washed three times. A purified protoplast suspension was subsequently obtained by adding 150 μL of 0.6 M mannitol as a stabilizing solution. Finally, the protoplast suspension was evenly spread onto tryptone–yeast extract–dextrose agar (TB3 agar) plates to screen for mononuclear protoplasts. All strains were preserved at the Edible Fungal Germplasm Resources Management Center of Fujian Province, Fuzhou, China [26].

### 2.2. Culture Conditions

The strains were inoculated onto potato dextrose agar (PDA) and incubated at 25 °C to maintain viability and obtain sufficient mycelial growth. Two heterokaryotic strains, Ag.c0002 and Ag.c0067, were separately inoculated into cultivation substrates. During cultivation, the temperature was maintained at 25 °C throughout the mycelial growth phase. For fruiting body induction, the temperature was reduced to 19 °C, and the relative humidity was maintained at approximately 95%.

### 2.3. Genome Sequencing

The monokaryotic strain Ag.c0002-1 was cultured on PDA. Fresh mycelium was collected and submitted to Wuhan Fisha Gene Information Co., Ltd. (Wuhan, Hubei, China) for whole-genome sequencing. Third-generation sequencing was performed using the Oxford Nanopore Technologies (ONT) platform, and a Hi-C library was constructed with the Illumina HiSeq platform (Illumina, San Diego, CA, USA). Second-generation sequencing was also carried out on the Illumina HiSeq platform.

### 2.4. Genome Assembly

De novo assembly of ONT long reads was performed using Flye (v2.5). The resulting assembly was further polished and corrected with Pilon (v1.23) using next-generation sequencing (NGS) data, yielding the final assembly [27].

Raw Hi-C sequencing reads were filtered and quality-checked using fastp and HiCUP. The resulting clean reads were then processed with the Juicer program to generate a genome fragment interaction matrix prior to scaffolding. This matrix was used as input for the 3d-DNA program for chromosome assembly. The resulting assembly was subsequently polished with the Juicebox program to obtain the final chromosome-level genome. To evaluate the quality of the *C. chaxingu* genome assembly, HiCPlotter software (v2.0) was employed to visualize chromosome interaction patterns, and genome completeness was evaluated using the fungi_odb10 database from BUSCO (v6.0.0) [28].

### 2.5. Repeat Sequence Annotation

Transposable elements (TEs) in the *C. chaxingu* genome were identified using RepeatMasker (v4.0.9) in combination with the Repbase TE library. In parallel, a de novo repeat library for the *C. chaxingu* genome was constructed using RepeatModeler (https://github.com/Dfam-consortium/RepeatModeler, accessed on 5 September 2024) to comprehensively build, refine, and classify consensus models of putative dispersed repeats [29,30,31]. In addition, long terminal repeat (LTR) retrotransposons were identified de novo using LTR-FINDER (v1.0.7), LTR-harvest (v1.5.11), and LTR-retriever (v2.7). Tandem repeats were detected using the Tandem Repeat Finder (TRF) software (v4.09), and simple sequence repeats (SSRs) were identified using the MISA (v1.0) package [32,33,34]. Finally, the libraries obtained from these approaches were merged, and RepeatMasker was used to identify and annotate the repeat content in the genome.

### 2.6. Non-Coding RNA Annotation

Transfer RNA (tRNA) genes were predicted using tRNAscan-SE (v1.3.1) with default parameters, while ribosomal RNA (rRNA) genes were identified using RNAmmer (v1.2) using the parameters -S euk -m lsu,ssu,tsu [35,36]. In addition, microRNAs (miRNAs) and small nuclear RNAs (snRNAs) were identified using cmscan (v1.1.2) in combination with the Rfam (v14.0) database with default parameters [37].

### 2.7. Protein-Coding Gene Structure Annotation

First, based on protein sequences from closely related species (*Agrocybe aegerita*) and transcript sequences obtained from third-generation full-length transcriptome data of the species, protein and transcript sequences were aligned to our genome assembly; protein-coding genes were then predicted using MAKER (v2.31.10) with default parameters [38]. Next, Augustus (v3.3.3) was trained to generate a species-specific model, which was subsequently used as input for de novo gene structure prediction [39]. Finally, the gene models obtained from these two approaches were integrated using EvidenceModeler (v2.1.0) (EVM) [40].

### 2.8. Protein Function Annotation 

Protein functions were inferred using BLASTP (NCBI BLAST v2.6.0+) and the Kyoto Encyclopedia of Genes and Genomes (KEGG) database with an E-value threshold of 1 × 10^−5^, based on the best hits against the National Center for Biotechnology Information (NCBI) Non-Redundant (NR), Translated European Molecular Biology Laboratory (TrEMBL), Eukaryotic Clusters of Orthologous Groups (KOG), and Swiss-Prot protein databases. Additionally, protein domains were annotated using PfamScan (v1.6) with the Pfam database from InterPro. Gene Ontology (GO) terms for each gene were assigned using Blast2GO (v6.0).

### 2.9. Preparation of Transcriptome Sequencing Samples

**Figure 1:**
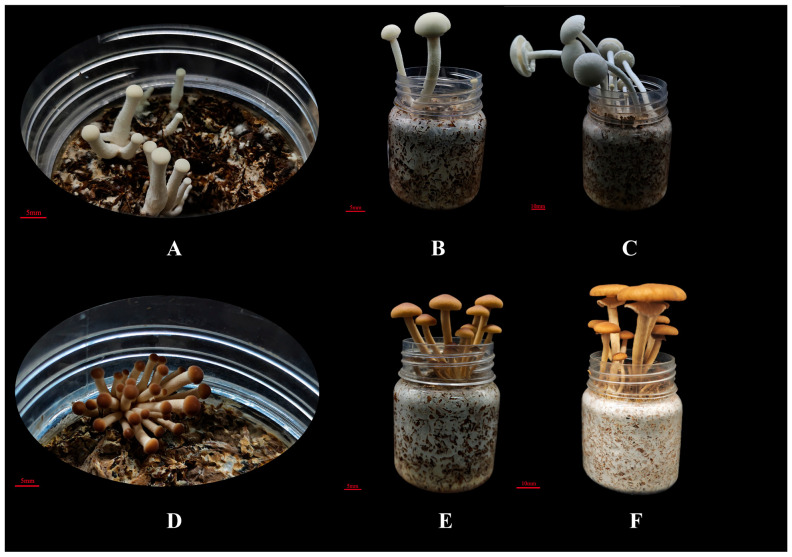
Different developmental stages of fruiting bodies in strains Ag.c0002 and Ag.c0067. (**A**–**C**) primordium, elongation, and mature stages of the fruiting body of strain Ag.c0002; (**D**–**F**) primordium, elongation, and mature stages of strain Ag.c0067.

Fruiting bodies of the brown strain Ag.c0067 and the white strain Ag.c0002 were cultivated. Based on their growth and developmental patterns, the fruiting bodies were classified into three stages: the primordium stage, the elongation stage, and the mature stage [41] (Figure 1). Pileus epidermis samples were collected from each stage (Table S1) and stored at −80 °C for long-term use. Among these, the pileus in the primordium stage was designated as YP, the pileus in the elongation stage was designated as EP, and the pileus in the mature stage was designated as MP.

### 2.10. Observation of Fruiting Body Pilei Phenotypes

The size of mushroom pilei at three distinct developmental stages was measured with a vernier caliper, and the color at each stage was determined using a handheld colorimeter. Prior to measurement, the colorimeter was calibrated using A4 white paper as a reference. The instrument was positioned over the central area of each mushroom pileus, and the corresponding L*, a*, b*, ΔL*, Δa*, and Δb* values were recorded. L* indicates lightness, ranging from 0 (black) to 100 (white). a* represents redness, ranging from −128 (green) to +128 (red), and b* reflects yellowness, ranging from −128 (blue) to +128 (yellow). ΔL*, Δa*, and Δb* represent the differences between the L*, a*, and b* values of the fruiting body and those of the A4 white reference paper [25,42].

### 2.11. Transcriptome Sequencing and Analysis

The collected samples were sent to Wuhan Fesha Gene Information Co., Ltd. (Wuhan, Hubei, China) for transcriptome sequencing, which was performed using the Illumina HiSeq platform. The Ag.c0002-1 genome assembled in this study was used as the reference genome. Transcriptome clean reads were aligned to the reference genome using HISAT2 (v2.2.1) [43]. Raw read counts were calculated using featureCounts (v2.0.3) and normalized to FPKM values using TBtools (v2.390) [44,45]. DESeq2 was employed to identify differentially expressed genes (DEGs) between developmental stages based on raw read counts, with thresholds set at |log2(fold change)| ≥ 1 and false discovery rate (FDR) < 0.05 [46]. GO and KEGG enrichment analyses of DEGs were performed in R (classic Fisher test, *p* ≤ 0.05). Additionally, intersection analysis was conducted using the online platform OmicStudio (https://www.omicstudio.cn/tool/6, accessed on 5 September 2024).

### 2.12. RT-qPCR Validation

Total RNA was extracted from the pileus tissues of the mushroom strains Ag.c0002 and Ag.c0067 at the primordium, elongation, and mature stages using the OMEGA E.Z.N.A. Plant RNA Kit (Omega Bio-Tek, Norcross, GA, USA), following the instructions from the manufacturer. The extracted RNA was subsequently used for validation.

The extracted RNA samples were quantified using a microplate reader. Complementary DNA (cDNA) was synthesized from the RNA using the Evo M-MLV Reverse Transcription Reagent Kit (with gDNA removal reagent for qPCR) Version 2 (AG Aikrui Bio, Changsha, China). Based on the selected candidate genes, primers for RT-qPCR were designed using Clone Manager Suite 7 software. The primer sequences are listed in Table 1.

cDNA was prepared according to the protocol provided with the SYBR Green Pro Taq HS Pre-mixed qPCR Kit (AG Aikrui Bio, China). qPCR amplification and detection were performed using the CFX96™ Real-Time System.

### 2.13. Data Analysis

Statistical analyses of pileus size and RT-qPCR data were performed using GraphPad Prism (v10.6.1). Significance levels were denoted as follows: ns, not significant; * *p* < 0.05; ** *p* < 0.01; *** *p* < 0.005. Colorimetric data were analyzed using Origin (v2021).

## 3. Results and Discussion

### 3.1. High-Quality Genome Assembly

ONT and Hi-C sequencing were employed to obtain genomic data for the mononuclear strain Ag.c0002-1, which were then used for genome assembly. ONT sequencing generated approximately 37.27 Gb of data, comprising 742,791 reads with an average read length of 50,176 bp and an N50 of approximately 0.05 Mb. Hi-C sequencing produced 25.45 Gb of data with an average read length of 150 bp.

De novo assembly based on the ONT sequencing data yielded a 51.7 Mb genome with an N50 of 4.25 Mb and a GC content of 51.06% (Table S2). Subsequently, the Hi-C data were used to anchor the preliminary assembly to chromosomes, resulting in 13 assembled chromosomes (Table S3) with an anchoring rate of 99.14%.

**Figure 2:**
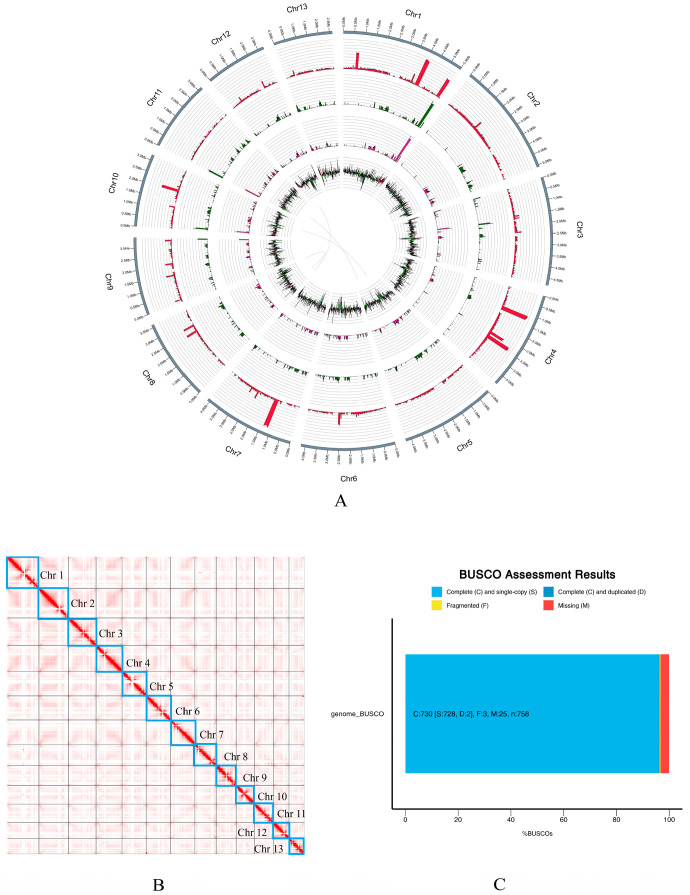
Whole-genome assembly of *C. chaxingu* strain Ag.c0002-1. (**A**) Characterization of the *C. chaxingu* genome. From outer to inner circles: chromosome representation, gene density, transposon density, repetitive sequence density, and GC content; (**B**) Hi-C interaction map of the *C. chaxingu* genome, with blue squares indicating chromosome lengths; (**C**) BUSCO assessment results.

The Hi-C interaction heatmap showed clear boundaries between chromosomes, with strong and continuous diagonal signals that indicated well-defined linear relationships within chromosomes. No significant misassemblies or deletions were detected. Interchromosomal interactions were weak, and no abnormal cross-interactions were observed, confirming accurate chromosome positioning. The overall genome structure was regular, with no large gaps or orientation errors, indicating a high-quality assembly with reliable chromosome-level accuracy (Figure 2B).

BUSCO assessment further demonstrated high genome completeness, with a completeness score of 96.3% (Figure 2C). The total size of the *C. chaxingu* Ag.c0002-1 genome assembly was approximately 51.7 Mb, with most sequences successfully anchored to 13 chromosomes (Figure 2A and Table 2). A total of 11,332 coding genes were predicted, with an average length of 2701 bp. In total, 17,299 proteins were predicted, with an average length of 473 amino acids (aa) and a GC content of 54.22% within coding sequences (CDSs).

### 3.2. Genome Composition Analysis

Repetitive sequences in the genome were identified using the method described in Section 2.5, resulting in a total of 10,710,773 bp of repetitive sequences, accounting for 20.71% of the genome (Table 3). Further classification revealed that these repeats were primarily composed of LTR elements, Long Interspersed Nuclear Elements (LINEs), and DNA elements. Analysis of repeat types identified 10,293,614 bp of interspersed repeats, representing 19.91% of the genome. The lengths of LTR elements and LINEs accounted for 7.01% and 1.80% of the genome, respectively, while DNA elements accounted for 0.15%. No SINEs were detected. In addition, simple repeats accounted for 0.78%, whereas low-complexity sequences and unclassified repeats accounted for 0.11% and 10.95%, respectively.

Additionally, non-coding gene prediction identified 180 tRNA genes with a total length of 14,850 bp, accounting for 0.029% of the genome, and 30 rRNA genes.

### 3.3. Functional Annotation of the Genome

**Figure 3:**
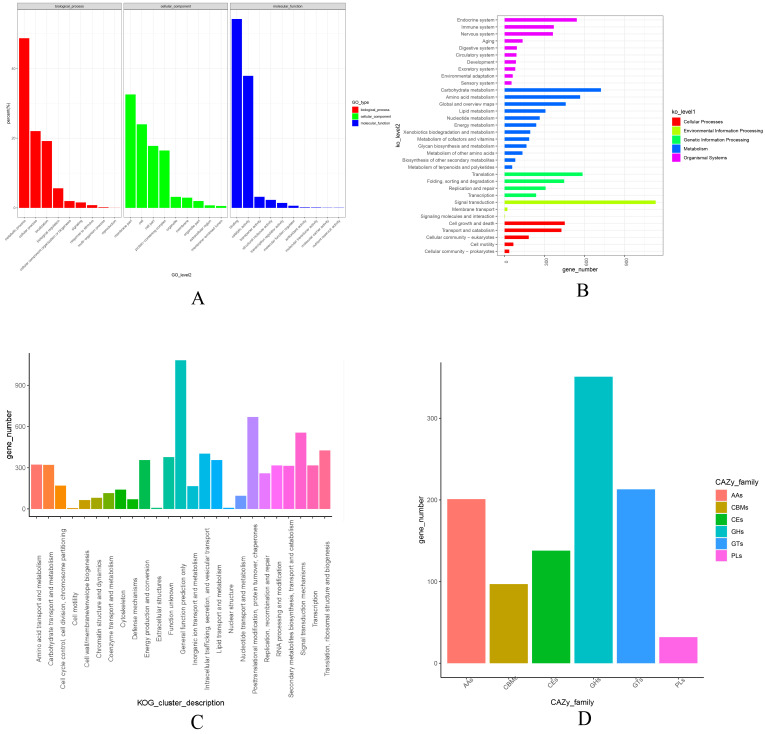
Functional Annotation of *C. chaxingu* Ag.c0002-1. (**A**) GO classification; (**B**) KEGG classification; (**C**) KOG classification; (**D**) CAZy classification.

A total of 16,573 functional proteins were identified in the *C. chaxingu* genome through annotation and comparison (Table 4 and Figure 3). Among them, 6885 proteins were assigned GO terms, with enrichment observed across the three major categories: biological process, cellular component, and molecular function. KEGG annotation assigned 5612 proteins to five major pathway categories: Cellular Processes, Environmental Information Processing, Genetic Information Processing, Metabolism, and Organismal Systems. The KOG database matched 6979 proteins, most of which were predicted to have general functional roles. The COG (Clusters of Orthologous Groups of proteins) database identified 4515 proteins, with the largest group associated with carbohydrate transport and metabolism. In addition, Pfam, Swiss-Prot, TrEMBL, and NR identified 10,935, 8581, 16,446, and 16,538 proteins, respectively. Furthermore, alignment against the CAZy (Carbohydrate-Active enZYmes Database) database identified 976 carbohydrate-active enzymes, including auxiliary activities (AAs), glycoside hydrolases (GHs), glycosyltransferases (GTs), polysaccharide lyases (PLs), carbohydrate esterases (CEs), and carbohydrate-binding modules (CBMs).

### 3.4. Comparative Genomic Analysis of Ag.c0002-1 and Other C. chaxingu Genomes

Compared with the genomes of strains AAE3, AC9, and MG21 (Table 5), the Ag.c0002-1 genome assembled in this study achieved a preliminary chromosome-level assembly. It contained fewer contigs and a larger contig N50, indicating higher assembly continuity and more complete long-read coverage. Compared with the two AS-5 genomes of mononuclear strains, the Ag.c0002-1 genome was anchored to 13 chromosomes. The two genomes showed similar sizes and contig N50 values, confirming the reliability of the assembled Ag.c0002-1 genome. This genome can serve as a reference for subsequent analyses.

### 3.5. Phenotypic Analysis of the Pileus of the C. chaxingu Fruit Body

**Figure 4:**
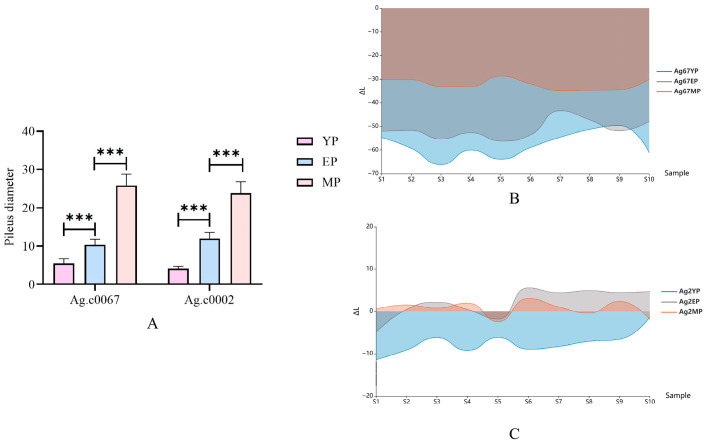
Analysis of the Pileus of the *C. chaxingu* Fruit Body. (**A**) Bar chart of pileus sizes at three developmental stages for the brown strain Ag.c0067 and the white strain Ag.c0002, *** indicates a highly significant difference between groups (*p* < 0.001); (**B**) lightness difference (ΔL) between the brown strain Ag.c0067 and A4 white paper at different developmental stages; (**C**) lightness difference (ΔL) between the white strain Ag.c0002 and A4 white paper at different developmental stages.

During the growth and development of the fruiting bodies of the white strain Ag.c0002 and the brown strain Ag.c0067, changes in pileus size were observed across the primordium (YP), elongation (EP), and mature (MP) stages. The pilei of both strains progressively enlarged during development (Figure 4A). Specifically, the average pileus size was approximately 4–6 mm at the primordium stage, increased to around 10–12 mm during the elongation stage, and reached about 20–30 mm at the mature stage. The results indicated that the rate of pileus expansion from the elongation to the mature stage was significantly higher than that from the primordium to the elongation stage.

The pileus color of the brown strain Ag.c0067 gradually lightened during development, whereas that of the white strain Ag.c0002 showed no visually perceptible change (Figure 1). However, measurements obtained using a handheld colorimeter revealed that, along the lightness (L-value) axis, the pilei of the white strain Ag.c0002 were darker at the primordium stage and gradually became lighter during the elongation and mature stages. Along the red-green axis (a-value), the pilei showed no significant changes across stages, whereas along the yellow-blue axis (b-value), the yellow hue gradually decreased during development. For the brown strain Ag.c0067, pileus brightness (L-value) increased progressively throughout development. Along the red-green axis (a-value), the red hue gradually decreased, while along the yellow-blue axis (b-value), the color remained stable across all stages (Figure 4 and Figure S1).

### 3.6. Transcriptome Analysis Results

**Figure 5:**
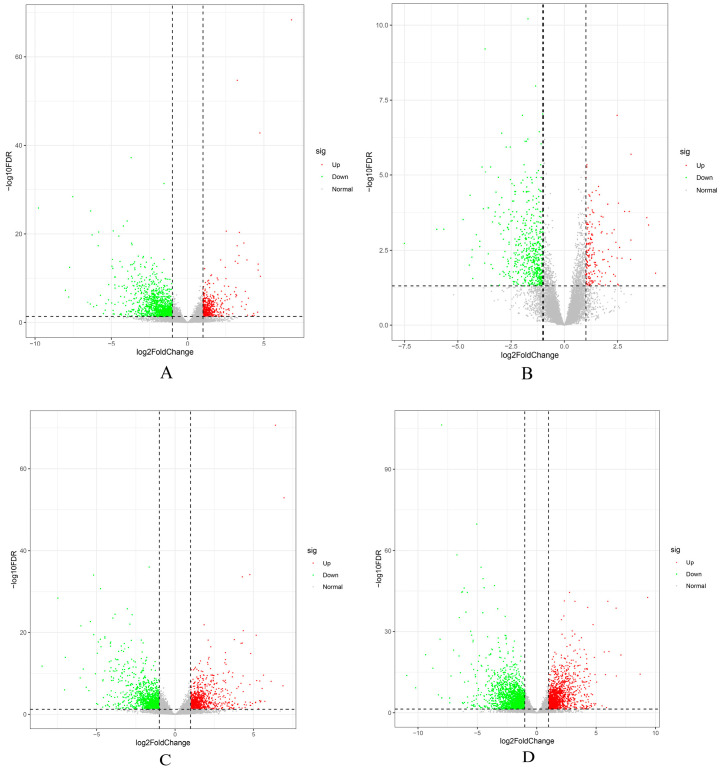
Volcano plot showing differentially expressed genes across developmental stages between the brown strain Ag.c0067 and the white strain Ag.c0002. (**A**–**D**) Volcano plots showing differentially expressed genes for the following pairwise comparisons: (**A**) Ag67YP_vs_Ag67EP; (**B**) Ag67EP_vs_Ag67MP; (**C**) Ag2YP_vs_Ag2EP; (**D**) Ag2EP_vs_Ag2MP.

Based on phenotypic analysis of the pileus of *C. chaxingu*, this study conducted comparative transcriptomic analyses of samples collected from different developmental stages at the center of the pileus to examine the expression patterns of genes involved in pileus expansion and color changes, and to identify candidate genes potentially regulating pileus growth and development (Figure 5). In the brown strain Ag.c0067, 1786 differentially expressed genes (DEGs) were identified between the primordium and elongation stages (Ag67YP_vs_Ag67EP), including 547 up-regulated genes and 1239 down-regulated genes. Between the elongation and mature stages (Ag67EP_vs_Ag67MP), 735 DEGs were identified, comprising 184 up-regulated and 551 down-regulated genes. For the white strain Ag.c0002, from the primordium to elongation stages (Ag2YP_vs_Ag2EP), 2033 DEGs were identified, including 924 up-regulated and 1109 down-regulated genes. Between the elongation and maturation stages (Ag2EP_vs_Ag2MP), 3506 DEGs were identified, comprising 1577 up-regulated and 1929 down-regulated genes.

### 3.7. Omics Analysis of Genes Associated with Pileus Growth and Development in the C. chaxingu

**Figure 6:**
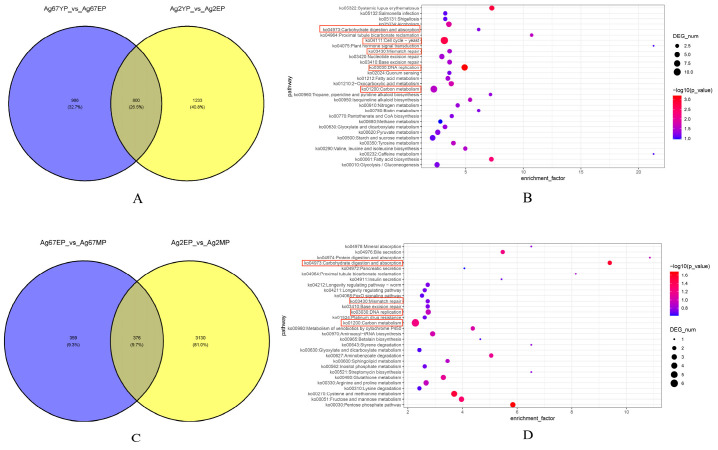
Venn diagram and KEGG enrichment bubble chart of differentially expressed genes common to the brown strain Ag.c0067 and the white strain Ag.c0002 across various developmental stages. (**A**) Venn diagram of the Ag67YP_vs_Ag67EP and Ag2YP_vs_Ag2EP comparisons; (**B**) KEGG enrichment bubble chart for the Ag67YP_vs_Ag67EP and Ag2YP_vs_Ag2EP comparisons; (**C**) Venn diagram of the Ag67EP_vs_Ag67MP and Ag2EP_vs_Ag2MP comparisons; (**D**) KEGG enrichment bubble chart for the Ag67EP_vs_Ag67MP and Ag2EP_vs_Ag2MP comparisons.

To identify candidate genes associated with pileus growth and development in *C. chaxingu*, intersection analyses were performed on the comparative transcriptomic results of the brown strain Ag.c0067 and the white strain Ag.c0002 at different developmental stages. In the intersection analysis of Ag67YP_vs_Ag67EP and Ag2YP_vs_Ag2EP, a total of 800 DEGs were identified (Figure 6A); In the intersection analysis of Ag67EP_vs_Ag67MP and Ag2EP_vs_Ag2MP, 376 DEGs were identified (Figure 6C).

KEGG enrichment analysis of DEGs during the primordium and elongation stages revealed significant enrichment in pathways such as DNA replication (ko03030), cell cycle—yeast (ko04111), and mismatch repair (ko03430) (Figure 6B). Further analysis indicated that these DEGs are involved in DNA repair, chromosome condensation during the cell cycle, and the initiation, elongation, and termination of DNA replication. In-depth mining of these DEGs identified multiple candidate genes potentially regulating pileus growth and development (*Chr10_00293*, *Chr1_00363*, *Chr2_00530*, *Chr1_00004*, *Chr8_00129*, and *Chr13_00163*). Among these genes, the protein encoded by *Chr10_00293* contains a DNA polymerase type-B epsilon subfamily catalytic domain, which plays a crucial role in DNA replication and synthesis; *Chr1_00363* encodes replication factor A protein 1 (RPA1), an essential subunit of the RPA complex, RPA participates in telomere maintenance by binding to telomeric G-quadruplexes (G4s) and promotes high-fidelity DNA replication and repair through acetylation; *Chr2_00530* encodes DNA ligase 1 that functions in coordination with DNA polymerase β to perform base excision repair of single-base lesions; *Chr1_00004* encodes proliferating cell nuclear antigen (PCNA), a cofactor of DNA polymerase δ, which interacts with multiple replication-related proteins to coordinate the DNA replication process; *Chr8_00129* encodes the chromosome condensation complex Condensin, subunit D2, previous studies have shown that this subunit can interact with other proteins to regulate gene expression; *Chr13_00163* encodes minichromosome maintenance proteins (MCMs), among which MCM2–7 constitute the core of the replicative DNA helicase [47,48,49,50,51,52,53,54]. The proteins encoded by these candidate genes are essential for the proper progression of DNA replication, which provides the genetic basis for cell proliferation and pileus development. In the intersection analysis between the elongation and maturation stages, DEGs were also significantly enriched in pathways such as DNA replication (ko03030) and mismatch repair (ko03430) (Figure 6D). Compared with the primordium-to-elongation transition, fewer DEGs were identified, and the diversity of encoded proteins decreased.

During the development of fruiting bodies from the primordium stage to maturity, the expression levels of most DEGs associated with DNA replication decreased. Specifically, compared with the primordium stage, the expression levels of genes encoding proteins such as DNA ligase I and replication factor A protein 1 (RPA1) declined during the elongation stage. A similar decrease was observed when comparing the elongation stage with the mature stage, indicating that DNA replication-related genes are gradually downregulated as development proceeds. This is likely due to the fact that, during the primordium stage, the fruiting body undergoes rapid growth accompanied by active cell proliferation, resulting in high expression of DNA replication-related genes. As development proceeds, the fruiting body gradually enters the stages of morphological differentiation and reproductive growth, during which cell division activity declines, leading to reduced expression of DNA replication-related genes at the elongation and maturation stages.

Beyond cell proliferation processes (e.g., DNA replication and repair), the continuous supply of energy and carbon sources is also crucial for pileus development. KEGG enrichment analysis of DEGs from the two intersection analyses revealed significant enrichment in pathways such as carbohydrate digestion and absorption (ko04973) and carbon metabolism (ko01200). Further examination of these pathways showed that the DEGs were involved in carbohydrate metabolism, including glycolysis/gluconeogenesis, the pentose phosphate pathway, and the glyoxylate cycle. Through in-depth analysis of these genes, this study identified several candidate genes potentially regulating pileus growth and development (e.g., *Chr10_00485*, *Chr11_00281*, *Chr1_00684*, *Chr10_00327*, and *Chr3_01131*). Among these genes, *Chr10_00485* encodes the transaldolase B, which is a key enzyme in the non-oxidative branch of the pentose phosphate pathway and may influence the organismal growth, development, and morphology of the organism; *Chr11_00281* encodes choline dehydrogenase (CHDH), which is distributed in the inner mitochondrial membrane of eukaryotes and plays an important role in electron transfer processes within the respiratory chain; the gene *Chr1_00684* encodes the catalytic domain of α-amylase, which hydrolyzes starch into glucose to supply metabolic energy; the protein encoded by *Chr10_00327* functions as a magnesium-transporting ATPase that utilizes ATP hydrolysis to drive Mg^2+^ transport, magnesium ions are essential for protein synthesis and cellular energy homeostasis; *Chr3_01131* encodes malate synthase A, which is the core rate-limiting enzyme of the glyoxylate cycle [55,56,57,58]. The proteins encoded by these candidate genes may coordinate the catabolism of carbon sources and the utilization of energy by modulating ion gradients or regulating metabolic intermediates. Moreover, these proteins may contribute, to varying degrees, to the efficient digestion and absorption of carbohydrates, help maintain the stability of the carbon metabolic network, and ultimately convert carbon sources and energy into the substances and driving force required for pileus growth and development. Further expression analyses revealed that these candidate genes showed an overall upward trend during development. Considering the substantial demand for energy and structural components (e.g., cell wall polysaccharides and ergosterol) during the formation of *C. chaxingu* fruiting bodies, the sustained upregulation of these genes is likely to promote the accumulation of essential metabolites and support the energy metabolism required for pileus growth and development.

### 3.8. Omics Analysis of Genes Associated with Pileus Color in C. chaxingu

Based on the color changes observed during the development of the brown strain (Ag.c0067) and the white strain (Ag.c0002), this study analyzed not only the growth and development of the pilei but also differences in their color intensity.

**Figure 7:**
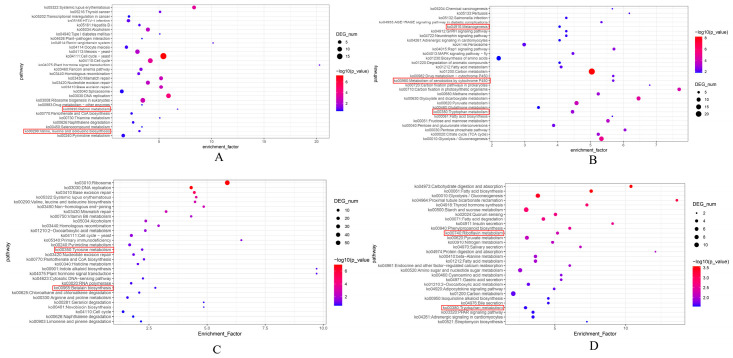
KEGG enrichment bubble chart of the brown strain Ag.c0067 and the white strain Ag.c0002 at different developmental stages. KEGG enrichment maps of genes in different comparisons: (**A**) upregulated genes in Ag67YP_vs_Ag67EP; (**B**) downregulated genes in Ag67YP_vs_Ag67EP; (**C**) upregulated genes in Ag2YP_vs_Ag2EP; (**D**) downregulated genes in Ag2YP_vs_Ag2EP.

Analysis of DEGs across the developmental stages of the brown strain Ag.c0067 yielded the following results. In the Ag67YP_vs_Ag67EP comparison, 168 upregulated genes were significantly enriched in 98 metabolic pathways, including the cell cycle—yeast (ko04111), DNA replication (ko03030), retinol metabolism (ko00830), valine, leucine, and isoleucine biosynthesis (ko00290), and other pathways (Figure 7A). In contrast, the 203 downregulated genes were significantly enriched in 242 metabolic pathways, notably carbon metabolism (ko01200), pyruvate metabolism (ko00620), glycolysis/gluconeogenesis (ko00010), tryptophan metabolism (ko00380), melanogenesis (ko04916), metabolism of xenobiotics by cytochrome P450 (ko00980), and other pathways (Figure 7B).

In the comparison between Ag67EP and Ag67MP, 67 upregulated genes were significantly enriched across 56 metabolic pathways, including tyrosine metabolism (ko00350), riboflavin metabolism (ko00740), aminoacyl−tRNA biosynthesis (ko00970), melanogenesis (ko04916), metabolism of xenobiotics by cytochrome P450 (ko00980), and other pathways (Figure S2A). A total of 123 downregulated genes were enriched across 147 pathways, including carbon metabolism (ko01200), biosynthesis of amino acids (ko01230), arginine and proline metabolism (ko00330), and other pathways. Additionally, several genes were also enriched in tyrosine metabolism (ko00350) and related pathways (Figure S2B).

Further analysis of KEGG enrichment results associated with pigment biosynthesis indicated that most candidate genes encoded proteins classified into the tyrosinase family or glutathione S-transferase (GST) family, such as *Chr8_00622* and *Chr3_00368*. Detailed examination revealed that, in the comparison between the primordium and elongation stages of the brown strain, most genes encoding GSTs showed an increasing expression trend. In contrast, in the comparison between the elongation and mature stages, most candidate genes encoding tyrosinases exhibited a decreasing expression trend.

Analysis of DEGs across the developmental stages of the white strain Ag.c0002 yielded the following results. In the Ag2YP_vs_Ag2EP comparison, 215 upregulated genes were enriched across 152 metabolic pathways, with significant enrichment in pathways such as ribosome (ko03010) and DNA replication (ko03030), as well as other pathways, including tyrosine metabolism (ko00350) and betalain biosynthesis (ko00965) (Figure 7C). A total of 115 downregulated genes were enriched across 238 metabolic pathways, with significant enrichment in carbohydrate digestion and absorption (ko04973), fatty acid biosynthesis (ko00061), riboflavin metabolism (ko00740), tryptophan metabolism (ko00380), and other pathways (Figure 7D).

In the Ag2EP_vs_Ag2MP comparison, 397 upregulated genes were enriched across 229 metabolic pathways, with significant enrichment in steroid biosynthesis (ko00100), DNA replication (ko03030), cell cycle—yeast (ko04111), and other pathways, including terpenoid backbone biosynthesis (ko00900) (Figure S2C). Meanwhile, 253 downregulated genes were enriched across 266 pathways, notably in fructose and mannose metabolism (ko00051), glutathione metabolism (ko00480), and other pathways (Figure S2D).

In-depth analysis of the KEGG enrichment results revealed that most candidate genes identified in the comparison between the primordium and elongation stages of the white strain belonged to the tyrosinase family (e.g., *Chr8_00622*, *Chr10_00596*) and the aldehyde dehydrogenase family 14 (e.g., *Chr4_01119*, *Chr4_01120*). Most tyrosinase-encoding genes exhibited a downregulated trend, whereas most aldehyde dehydrogenase-encoding genes exhibited an upregulated trend. In the comparison between the elongation and mature stages, the selected candidate genes primarily belonged to the cytochrome P450 family (e.g., *Chr2_00798*, *Chr6_00387*) and the GST family (e.g., *Chr3_01178*, *Chr6_00465*). Among these, cytochrome P450-encoding genes predominantly exhibited a downregulated trend, whereas genes encoding GSTs predominantly exhibited an upregulated trend.

In summary, through a comprehensive analysis of pileus samples from the brown strain Ag.c0067 and the white strain Ag.c0002 at different developmental stages, several candidate genes potentially involved in regulating pileus color intensity in *C. chaxingu* were identified. The proteins encoded by these genes predominantly belong to the tyrosinase families, GST, aldehyde dehydrogenase, and cytochrome P450 families. In the brown strain Ag.c0067, tyrosinase gene expression did not differ significantly between the primordium and elongation stages but decreased markedly from the elongation to the mature stage, consistent with phenotypic observations. In contrast, the white strain Ag.c0002 exhibited minimal pileus color changes, although color-difference analysis revealed increased brightness and reduced yellowness and blueness values. Given that tyrosinase is a key enzyme involved in melanin biosynthesis, these results indicate that tyrosinase genes likely participate in pigment formation and contribute to pileus color regulation in *C. chaxingu* [23,59]. Most GST genes exhibited an upward trend in expression during fruiting body development, with more GST genes identified in the brown strain than in the white strain. Previous studies in plants have shown that GSTs are essential for anthocyanin transport and storage, suggesting that they may similarly function in pigment transport in *C. chaxingu* [60,61]. Additionally, aldehyde dehydrogenase and cytochrome P450 genes were identified. Aldehyde dehydrogenase regulates pigment synthesis in Neurospora spp. and Fusarium spp. and participates in pigment formation during brown veil development in Lentinula edodes. Cytochrome P450 enzymes play key roles in pileus color inheritance in *Hypsizygus marmoreus* [22,62,63]. These findings indicate that aldehyde dehydrogenase and cytochrome P450 genes may also regulate pileus color formation in *C. chaxingu*.

### 3.9. RT-qPCR Validation

**Figure 8:**
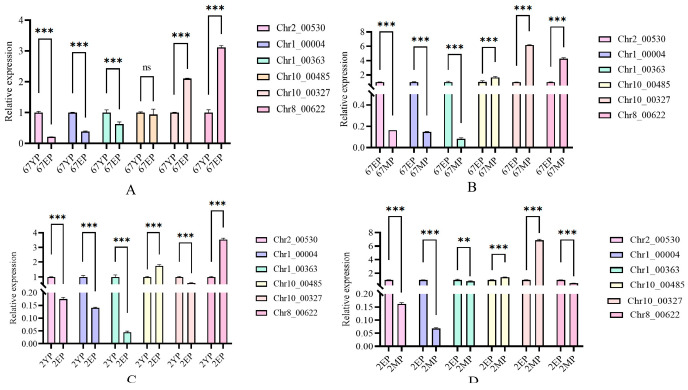
Relative expression levels of candidate genes across different developmental stages, Where ***, **, and ns indicate extremely significant differences (*p* < 0.001), significant differences (*p* < 0.01), and no statistically significant difference (*p* ≥ 0.05), respectively. Relative expression levels of candidate genes at different developmental stages: (**A**) brown strain Ag.c0067, primordium vs. elongation stages; (**B**) brown strain Ag.c0067, elongation vs. maturation stages; (**C**) white strain Ag.c0002, primordium vs. elongation stages; (**D**) white strain Ag.c0002, elongation vs. maturation stages.

Based on the above analyses, GAPDH was used as the housekeeping gene for RT-qPCR validation of the candidate genes. The results (Figure 8) show that most candidate genes exhibited expression trends consistent with the transcriptome analysis.

## 4. Conclusions

This study successfully assembled the genome of Ag.c0002-1, a white variant of the mushroom *C. chaxingu*, which can provide an important reference gene resource for subsequent research on the multi-trait genetics of *C. chaxingu*. Based on in-depth analysis of the genome and transcriptome data, this study has further deepened the scientific understanding of the molecular mechanisms of pileus development and color formation in *C. chaxingu*, while providing crucial theoretical support and candidate genes for improving its commercial traits. Specifically, pileus size and color are core traits affecting the commercial value of *C. chaxingu*. The cell division and carbon metabolism-related genes (associated with pileus size regulation) and pigment metabolism-related genes (associated with color formation) identified in this study may potentially serve as important targets for molecular breeding. Overall, these findings establish a solid foundation for the targeted breeding of new *C. chaxingu* varieties with improved pileus morphology and colors that better meet consumer preferences.

## Figures and Tables

**Table 1:** RT-qPCR Primer Sequences.

Gene Name	Primer	Primer Sequences (5′–3′)
*glyceraldehyde-3-phosphate dehydrogenase*	qgpd-F	TCATCAATGGCAAGCCTG
	qgpd-R	CCAAGTTCACACCACAGACG
*Chr2_00530*	q2-530F	TTCATGGGCCGTCAGCAAAC
	q2-530R	TCGCATCTTCTCCTGCTTTC
*Chr1_00004*	q1-4F	CGTGACAGACGCGAACTTTG
	q1-4R	TAAAGGCCGGCGCTGAGAAC
*Chr1_00363*	q1-363F	ATACAGAGCACACCGTACAG
	q1-363R	AGCATGGCCTGCATGTAGTG
*Chr10_00485*	q10-485F	AAGAGCTGATTGCCCTCTAC
	q10-485R	ATACCATCGTCGCGCTCCAG
*Chr10_00327*	q10-327F	ACCTCTCGTTGCACGGAAAG
	q10-327R	CGATACTCGCCGCTAAGAGG
*Chr8_00622*	q8-622F	TGGCTCTACCATCCAGAAAG
	q8-622R	CGGCGTCGTTCAAAGTTCTC

**Table 2:** Statistical Results of the *C. chaxingu* Genome Assembly.

Assembly Feature	*C. chaxingu*
Number of Chromosome	13
Assembly size (Mb)	51.7
BUSCO (%)	96.3%
Average gene length (bp)	2701
Gene number	11,332
Protein number	17,299

**Table 3:** Annotation of Repetitive Sequences in the Genome of Ag.c0002-1 (*C. chaxingu*).

Class	Length (bp)	Percent (%)
bases masked	10,710,773	20.71
Total interspersed repeats	10,293,614	19.91
LINEs	518,932,889	1.80
LTR elements	14,653,623,878	7.10
SINEs	0	0
DNA elements	6,577,049	0.15
Unclassified	59,565,659,798	10.95
Simple repeats	6,108,403,425	0.78
Low complexity	104,757,492	0.11

**Table 4:** Genome Annotation of Ag.c0002-1 (*C. chaxingu*).

Database	Annotated Number	Annotated Ratio (%)
GO	6885	39
COG	4515	26
KEGG	5612	32
KOG	6979	40
NR	16,538	95
Pfam	10,935	63
Swiss prot	8581	49
TrEMBL	16,446	95
Total	16,573	95

**Table 5:** Comparative Genomic Analysis of Ag.c0002-1 and Other *C. chaxingu* Genomes.

Assembly Feature	*A. cylindracea* (Ag.c0002-1)	*A. aegerita* AAE3	*A. cylindracea* AC9	*A. cylindracea* MG21	*A. chaxingu* AS-5 (CchA)	*A. chaxingu* AS-5 (CchB)
Assembly size (Mb)	51.7	44.8	56.5	56.3	50.6	51.7
Number of contigs	42	2668	4609	11,621	-	-
Contig N50 (kp)	3050.54	134.3	86	22.1	3950	3970
Number of scaffolds	-	122	3790	10,976	-	-
Scaffold N50 (kp)	-	768.4	547.3	23.2	-	-
GC percent (%)	51.06	51	51	51	50.99	50.98
Number of Chromosome	13	-	-	-	13	13
Number of predicted gene models	11,332	14,110	15,384	22,535	14,376	14,207
Assembly level	Chromosome	Scaffold	Scaffold	Scaffold	Chromosome	Chromosome
Reference	This study	[13]	[11]	[12]	[14]	[14]

## Data Availability

The original data presented in the study are openly available in the National Center for Biotechnology Information Sequence Read Archive at accession number PRJNA1399432 (https://dataview.ncbi.nlm.nih.gov/object/PRJNA1399432, accessed on 5 January 2026) and PRJNA1400552 (https://dataview.ncbi.nlm.nih.gov/object/PRJNA1400552, accessed on 5 January 2026).

## Supplementary Materials

The following supporting information can be downloaded at: https://www.mdpi.com/article/10.3390/jof12010063/s1, File S1, Table S1: Fruiting body specimens of strains Ag.c0002 and Ag.c0067 at different developmental stages; Table S2: Whole-Genome Assembly Statistics of the Mononuclear Strain Ag.c0002-1; Table S3: Chromosome assembly results of strain Ag.c0002-1. File S2, Figure S1: Phenotypic Analysis of the Pileus of the *C. chaxingu* Fruit Body; Figure S2: KEGG enrichment bubble chart of the brown strain Ag.c0067 and the white strain Ag.c0002 at different developmental stages.
