# Supplementary material for: Genome and Comparative Transcriptome Analysis of Growth and Developmental Changes in the Pileus of the Cyclocybe chaxingu
Source: J Fungi (Basel). 2026 Jan 13;12(1):63. doi: 10.3390/jof12010063 (PMC12843369; doi:10.3390/jof12010063)
Supplement: Supplementary file 1 [file jof-12-00063-s001.zip › File S1.pdf]

**Table S1.** Fruiting body specimens of strains Ag.c0002 and Ag.c0067 at different developmental stages

| Ag.c0002 fruiting bodies at different developmental stages | Sample ID | Ag.c0067 fruiting bodies at different developmental stages | Sample ID |
|---|---|---|---|
| Pilei epidermis (primordium stage) | Ag2YP | Pilei epidermis (primordium stage) | Ag67YP |
| Pilei epidermis (elongation stage) | Ag2EP | Pilei epidermis (elongation stage) | Ag67EP |
| Pilei epidermis (mature stage) | Ag2MP | Pilei epidermis (mature stage) | Ag67MP |

**Table S2.** Whole-Genome Assembly Statistics of the Mononuclear Strain Ag.c0002-1

| Genome version | Sequence number | Total length (bp) | N50 (bp) | N90 (bp) | GC content (%) |
|---|---|---|---|---|---|
| All | 23 | 51,707,697 | 4,249,876 | 3,175,728 | 51.06 |
| Chromosome | 13 | 51,263,077 | 4,249,876 | 3,175,728 | 51.05 |

**Table S3.** Chromosome assembly results of strain Ag.c0002-1

| Chromosome | Chr. size (bp) | Contig number | Contig size (bp) | GC content (%) |
|---|---|---|---|---|
| Chr1 | 5,515,660 | 2 | 5,515,560 | 51.01 |
| Chr2 | 5,092,105 | 2 | 5,091,605 | 50.84 |
| Chr3 | 4,797,647 | 5 | 4,796,047 | 51.27 |
| Chr4 | 4,429,194 | 1 | 4,429,194 | 51.10 |
| Chr5 | 4,249,876 | 3 | 4,248,876 | 51.03 |
| Chr6 | 4,182,078 | 4 | 4,180,978 | 51.06 |
| Chr7 | 4,115,196 | 1 | 4,115,196 | 50.95 |
| Chr8 | 3,621,281 | 2 | 3,620,781 | 51.06 |
| Chr9 | 3,464,621 | 2 | 3,464,521 | 51.11 |
| Chr10 | 3,229,999 | 4 | 3,228,899 | 51.24 |
| Chr11 | 3,175,728 | 2 | 3,175,628 | 51.12 |
| Chr12 | 2,759,526 | 3 | 2,758,526 | 50.94 |
| Chr13 | 2,630,166 | 1 | 2,630,166 | 51.03 |

[S3] Chr. size indicates the size of the chromosome
