# Supplementary material for: Genome and Comparative Transcriptome Analysis of Growth and Developmental Changes in the Pileus of the Cyclocybe chaxingu
Source: J Fungi (Basel). 2026 Jan 13;12(1):63. doi: 10.3390/jof12010063 (PMC12843369; doi:10.3390/jof12010063)
Supplement: Supplementary file 1 [file jof-12-00063-s001.zip › File S2.pdf]

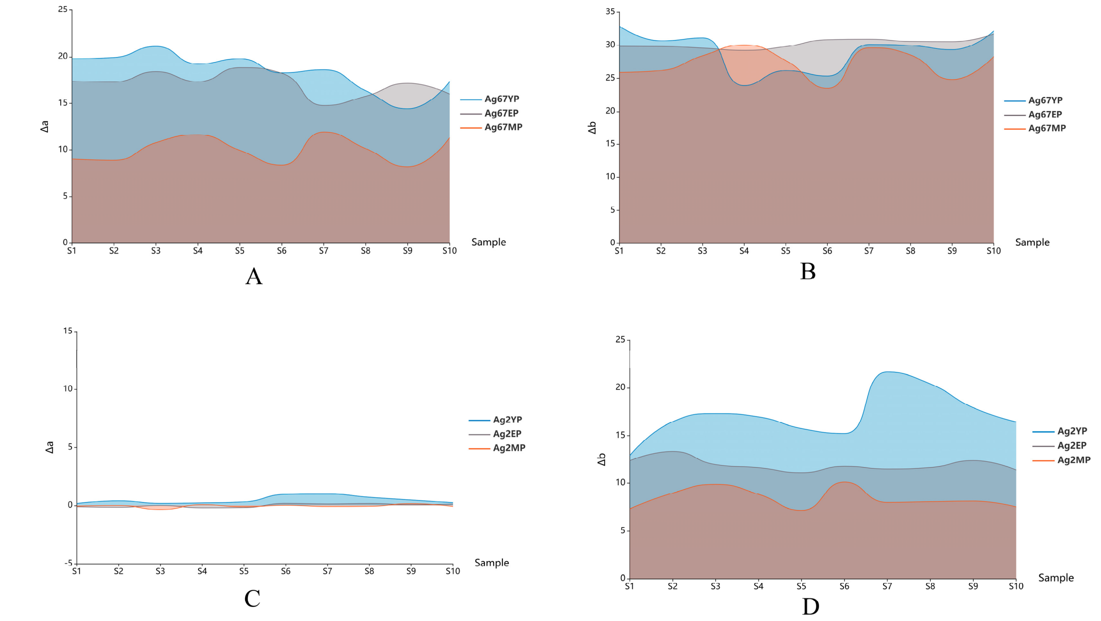

Figure S1. Phenotypic Analysis of the Pileus of the *C. chaxingu* Fruit Body. (A–B) show the red–green (Δa) and yellow–blue (Δb) differences between the brown strain Ag.c0067 at various developmental stages and A4 white paper; (C–D) show the corresponding differences for the white strain Ag.c0002

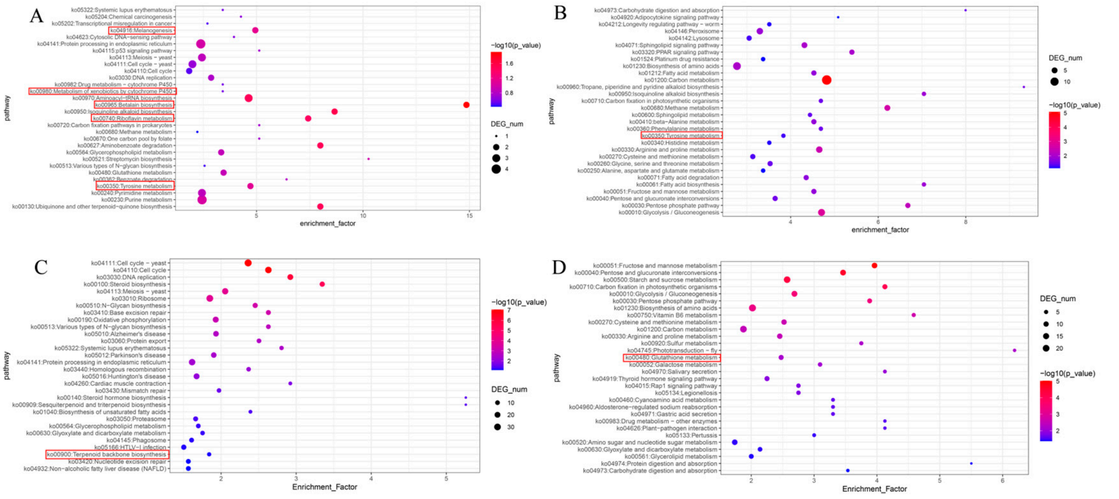

Figure S2. KEGG enrichment bubble chart of the brown strain Ag.c0067 and the white strain Ag.c0002 at different developmental stages. KEGG enrichment maps of genes in different comparisons: (A) upregulated genes in Ag67EP_vs_Ag67MP; (B) downregulated genes in Ag67EP_vs_Ag67MP; (C) upregulated genes in Ag2EP_vs_Ag2MP; (D) downregulated genes in Ag2EP_vs_Ag2MP
